# A Microarray-Based Analysis Reveals that a Short Photoperiod Promotes Hair Growth in the Arbas Cashmere Goat

**DOI:** 10.1371/journal.pone.0147124

**Published:** 2016-01-27

**Authors:** Bin Liu, Fengqin Gao, Jun Guo, Dubala Wu, Bayasihuliang Hao, Yurong Li, Cunfa Zhao

**Affiliations:** 1 Institute of Animal Husbandry, Academy of Agriculture and Stockbreeding Sciences, Hohhot, Inner Mongolia, China; 2 Kunming Institute of Animal Science, Chinese Academy of Sciences, Kunming, Yunna, Inner Mongolia, China; 3 Grassland Research Institute, Chinese Academy of Agricultural Sciences, Hohhot, China; 4 College of Life Science, Inner Mongolia Agricultural University, Hohhot, Inner Mongolia, China; 5 Etuokeqianqi Arctic God Research Institute of Cashmere and Livestock, Erdos, China; University of Alabama at Birmingham, UNITED STATES

## Abstract

Many animals exhibit different behaviors in different seasons. The photoperiod can have effects on migration, breeding, fur growth, and other processes. The cyclic growth of the fur and feathers of some species of mammals and birds, respectively, is stimulated by the photoperiod as a result of hormone-dependent regulation of the nervous system. To further examine this phenomenon, we evaluated the Arbas Cashmere goat (*Capra hircus*), a species that is often used in this type of research. The goats were exposed to an experimentally controlled short photoperiod to study the regulation of cyclic cashmere growth. Exposure to a short photoperiod extended the anagen phase of the Cashmere goat hair follicle to increase cashmere production. Assessments of tissue sections indicated that the short photoperiod significantly induced cashmere growth. This conclusion was supported by a comparison of the differences in gene expression between the short photoperiod and natural conditions using gene chip technology. Using the gene chip data, we identified genes that showed altered expression under the short photoperiod compared to natural conditions, and these genes were found to be involved in the biological processes of hair follicle growth, structural composition of the hair follicle, and the morphogenesis of the surrounding skin appendages. Knowledge about differences in the expression of these genes as well as their functions and periodic regulation patterns increases our understanding of Cashmere goat hair follicle growth. This study also provides preliminary data that may be useful for the development of an artificial method to improve cashmere production by controlling the light cycle, which has practical significance for livestock breeding.

## Introduction

In Inner Mongolia, the Cashmere goat (*Capra hircus*) is important for its wool. Cashmere is always used to make luxury knitted fabric; thus, it has great economic value. The hair of the Cashmere goat is mainly divided into two types: guard hair produced by primary hair follicles and under hairs (referred to commercially as cashmere) produced by secondary follicles [1]. The hairs are highly similar in structure and composition but have notable differences in the degree of fineness, which is determined by each follicle's size and shape. Cashmere production depends primarily on the area and length of the cashmere fiber, which are determined by the number of secondary follicles and the hair growth cycle [2]. Moreover, there is a large difference among the different varieties of Cashmere goat. In addition to genetic factors, environmental factors are also important, including photoperiod, nutrition, and livestock management. Nutritional conditions typically affect cashmere production and quality, and the seasonal photoperiod induces a large increase in cashmere production [3]. This seasonal phenomenon depends on the photoperiod-dependent regulation of the endocrine and the nervous systems by illumination, which can release a variety of hormones. Previous studies have shown that melatonin (MEL) [4–6], prolactin (PRL), growth hormone, insulin-like growth factor 1 (IGF-1), and thyroid hormone directly affect cashmere growth in Australian goats [7–12].

**Fig 1:**
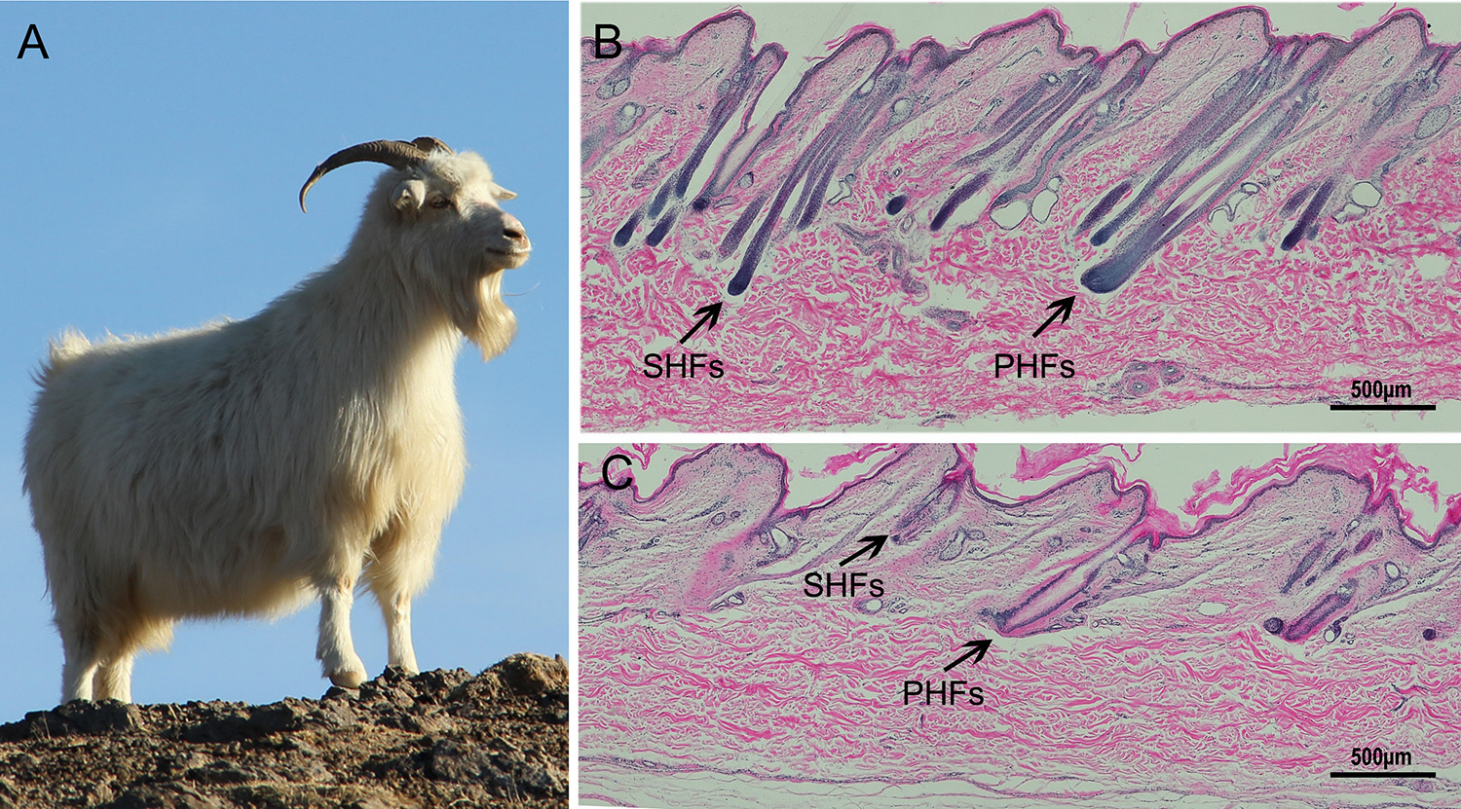
Photograph of white Arbas Cashmere goat and paraffin sections of Cashmere goat skin stained with hematoxylin & eosin. A. Photograph of a white Arbas Cashmere goat. B. Longitudinal section of goat skin sampled during the short photoperiod (anagen phase). C. Longitudinal section of goat skin sampled during the natural photoperiod (pro-anagen phase). The black arrows indicate the primary hair follicles (PHFs) and secondary hair follicles (SHFs) in the samples. Scale bars: 500 μm.

Arbas Cashmere goats (Fig 1A), a type of Inner Mongolian white Cashmere goat, are distributed in the western areas of Inner Mongolia, China, and their cashmere growth exhibits a strong seasonal variation [13] similar to that of Australian Cashmere goats. The activity of the secondary hair follicles (referred to as vellus hair) is regulated by various molecular signals with strict periodicity, which proceeds through the anagen, catagen, and telogen phases. In contrast, primary hair follicles do not have a clear telogen. Because the secondary hair follicles were activated relatively late in March, the cashmere grows out of the skin until July; thus, this stage should be named pro-anagen. This growth cycle of secondary hair follicles is related to the season; thus, the length of Cashmere goat fiber is strongly influenced by the season, and the inherent biological mechanism underlying this regulation is highly complex [14, 15]. The cashmere of Arbas begins to grow out of the skin from July to August, stops in the following January, and begins to molt at the end of April or early May when the new cashmere follicles are activated [13]. Exogen and kenogen have been identified and may be superimposed on telogen. In mice, the process of shedding hair shafts is called exogen. Kenogen means that the exhausted exogen hair actually sheds, and the hair follicle remains empty [16–18]. In the exogen/kenogen in Arbas Cashmere goat, the secondary hair follicles come into telogen in January until late March or early April; thus, their kenogen should occur from January to late March or early April. The secondary hair follicles come into anagen I-III, and exogen should not occur in the secondary hair follicles[19, 20].

Based on previous reports [21, 22], this phenomenon is likely caused by a series of hormones produced by the nervous system in the Cashmere goat following the short-day stimulation; these hormones affect the hair follicle and regulate cashmere growth. Currently, the genes related to the hair follicle growth cycles in humans and mice have been extensively studied, but the molecular mechanisms of the growth cycle of secondary hair follicles in the Cashmere goat remain unclear. In particular, there is no detailed evidence on how the photoperiod activates anagen or the types of genes that regulate this process.

With the development of next-generation sequencing technologies, digital data analysis provides us with more specific ways to identify differential gene expression. Therefore, changes in gene expression during the different stages of the hair follicle growth cycle can be detected at the transcript level, which provides us with a theoretical basis for further study of the cashmere growth mechanism [23–25].

Using gene chip technology for expression profiling, we filtered the differentially expressed genes between the artificial short-day photoperiod and natural conditions from the skin tissue of Arbas Cashmere goats. We discuss the differences in gene expression and their possible molecular biological roles in the two types of skin tissue to provide a further theoretical basis for research on the cashmere growth mechanism.

## Materials and Methods

### Ethics Statement

This study was performed in the Cashmere goat technology demonstration zone of Ordos City in Inner Mongolia, China. The goat skin tissue was collected in accordance with the International Guiding Principles for Biomedical Research Involving Animals and was approved by the Animal Ethics Committee of the Inner Mongolia Academy of Agriculture and Animal Husbandry Sciences, which is responsible for Animal Care and Use in the Inner Mongolia Autonomous Region of China. Skin excision was performed under xylazine hydrochloride anesthesia, and all efforts were made to minimize the goats' suffering.

### Animals and Tissues

Based on their similar weights, health conditions and good reproductive performance in a group of the same strain, we chose 6 female adult Arbas Cashmere goats that were 2 years old as our research specimens and randomly divided them into a test group (T1, T2, T3) and a control group (C1, C2, C3). The goats in the test group were grazed, fed, and allowed to drink from 9:30–16:30 daily, and they were housed in a dark shed with good air conditions for the remainder of the time. The temperature in the shed was 1°C colder than the outside temperature, and the opacity was controlled at approximately 0.1 lux. The control group grazed under natural conditions. We used regular management to care for all of the goats. The only factor that differed between the two groups was the photoperiod. Our experimental study was performed from May 2011 to May 2012. We obtained samples from one side of the body. After iodine disinfection and alcohol deiodination, we cut skin samples using sterile blades, quickly placed them into liquid nitrogen and stored them in a freezer (-80°C). A portion of the skin samples was used for gene chip analysis; the residual tissues were used to prepare paraffin sections, which were cut at 7 μm for hematoxylin-eosin staining and morphological observations.

### RNA extraction and microarray hybridization

TRIzol (Invitrogen, Gaithersburg, MD, USA) was used for the total RNA extractions according to the manufacturer's protocol. The quantity and quality of RNA were measured using a NanoDrop ND-1000 spectrophotometer. The mRNAs were purified with a NucleoSpin® RNA Clean-up Kit (MACHEREY-NAGEL, Germany). The concentrations of the RNAs present in the samples and the OD 260/280 ratios of the samples were determined using a NanoDrop ND-2000 spectrophotometer. The quality was assessed by standard denaturing agarose gel electrophoresis. Reverse transcription of first-strand cDNAs was performed using an oligo dT primer with T7 promoter sequences. The control group was labeled with Cy3, and the experimental group was labeled with Cy5. The samples were sent to CapitalBio Corporation (Beijing, China) for hybridization to an Agilent Sheep Gene Expression Microarray (Santa Clara, CA, USA).

Each RNA sample was hybridized to one microarray slide. The total RNA from each sample was amplified and transcribed into a fluorescent cRNA using the Agilent Quick Amp Labeling protocol (version 5.7, Agilent Technologies). The labeled cRNAs were hybridized onto the Whole Genome Oligo Array (4x44K, Agilent Technologies). This study used the G2519F sheep gene expression profile chip from Agilent, which is 8×15k in size and contains 15,208 sheep genes for expression profiling. The labeled DNA was dissolved in 80 μl of hybridization buffer (3× saline sodium citrate [SSC], 0.2% sodium dodecyl sulfate [SDS], 5× Denhardt's solution, and 25% formamide) overnight at 42°C. After the hybridization, the gene chips were washed for 5 min (0.2% SDS, 2× SSC) at 42°C and then washed for 5 min (0.2× SSC) at room temperature. After drying, an Agilent G2565CA Microarray Scanner was used to obtain the hybridization images of the gene chips.

### Microarray data analysis

Feature Extraction software was used to transform the image signal into a digital signal, and then, the original data were normalized (fold change ≥ 2 or ≤ 0.5) using the Lowess method (locally weighted scatterplot smoothing). If there were more than three biological replicates, the samples were analyzed with SAM (Significance Analysis of Microarrays) software to select the differentially expressed genes (fold change ≥ 2 or ≤ 0.5); chip hybridization was performed with the assistance of the Beijing CapitalBio Company.

### Real-time polymerase chain reaction

We validated the differences in gene expression between Cashmere goats raised in natural and artificial conditions using the real-time quantitative polymerase chain reaction (qPCR) technique. Based on the results of the gene microarray analysis, we selected three representative genes that had significant differences for qPCR (Table 1). Information on the primer design and the amplified genes is provided in Table 1. The RNA extraction was performed according to the method described in the gene microarray analysis. Glyceraldehyde 3-phosphate dehydrogenase (GAPDH) was used as an internal standard in the real-time PCR amplification. We used the SYBR Green I fluorescence method to quantify the relative expression of each gene. Triplicate samples were used, and the cycle threshold (Ct) values were converted into relative amplification levels. The differences in gene expression between the two samples were calculated using the 2^−ΔΔCt^ method.

**Table 1:** Primers for real-time polymerase chain reaction analysis of the differentially expressed genes.

Gene	GB. Accession		Primer Sequence 5'→3'	Amplicon
**CYP1A1**	NM_001129905.1	F	ATGACCACGATGACCAAGAGTT	271
		R	TTCTCGTCCAGCCTCTTATCC	
**keratin 34**	HQ283081.1	F	ATGTGCCACCACCAATGCTA	125
		R	ACCTTTCCTGGGCTTGTTCTA	
**MLN**	NM_001009439.1	F	TTTGTTCCCATCTTCACCTACG	108
		R	GCCCACCTCCTCTGACCTC	
**GAPDH**	NM_001034034	F	AAGTTCAACGGCACAGTCAA	125
		R	ACCACATACTCAGCACCAGC	

## Results

### Histological analysis of goat skin

To ensure the accuracy of the results, the histology samples were obtained from the same tissue used for the gene chip microarray analysis. Primary hair follicles are long, do not contain kinks, and develop during the first wave of hair follicle morphogenesis [26, 27]. The subsequent growth of hair during morphogenesis is the result of secondary hair follicles. Compared with primary hair follicles, dermal papilla of secondary hair follicles produce smaller hair shafts, which are thinner and show curvatures at varying degrees. In Cashmere goats, hard, rough wool is produced from primary hair follicles, and fine and soft cashmere is produced by secondary hair follicles. The tissue sections from the short photoperiod show that the quantities of the primary hair follicles were significantly increased and extended to the subcutaneous tissue, which was clearly in anagen phase (Fig 1B). Under the short photoperiod conditions, the Cashmere goat histology results showed that hair follicles have remarkable anagen morphological characteristics and are significantly thicker compared with the control group. Primary and secondary follicles grow significantly longer, and the diameter of the dermal papilla is greater, hair follicle density also increases significantly and is about 90% more than the control group (Table 2). Furthermore, the morphological structure of the inner root sheath is clearly visible, the hair follicle passageway extends from the dermal papilla to the skin, and the hair follicle cells are active. In contrast, the control group is still in the pro-anagen phase, with thinner skin, no significant internal root sheath, and smaller and fewer hair follicles. A large amount of the hair shaft was observed to have fallen off, leaving only empty hair follicle passageways (Fig 1C). Therefore, the histomorphology results clearly show that the short photoperiod can induce the hair follicles to enter the anagen phase.

**Table 2:** Skin thickness, length of the primary and secondary follicles, density of the primary and secondary follicles and diameters of the primary and secondary follicles.

Group	Short photoperiod	Natural photoperiod
**Skin thickness (μm)**	1826.80±91.44^A^	1651.68±182.23^B^
**Length of the primary follicles (μm)**	1654.15±164.68 ^A^	916.23±217.79 ^B^
**Length of the secondary follicles (μm)**	1039.54±224.37 ^A^	433.62±63.47 ^B^
**Diameter of the primary follicle dermal papilla (μm)**	152.79±25.15 ^A^	106.18±20.68 ^B^
**Diameter of the secondary follicle dermal papilla (μm)**	68.76±21.61 ^A^	45.51±10.46 ^B^
**Density of the primary follicles (·mm**^**-2**^**)**	57.66±7.60 ^A^	27.10±1.77 ^B^
**Density of the secondary follicles (·mm**^**-2**^**)**	6.49±0.10 ^A^	3.68±0.63 ^B^

A total of 30 H&E sections were examined under short and natural photoperiods. Means with different lowercase letters within the same row indicate significant differences between different columns. Means with different capital superscripts within the same row indicate highly significant differences between different columns.

### Hierarchical cluster analysis

**Fig 2:**
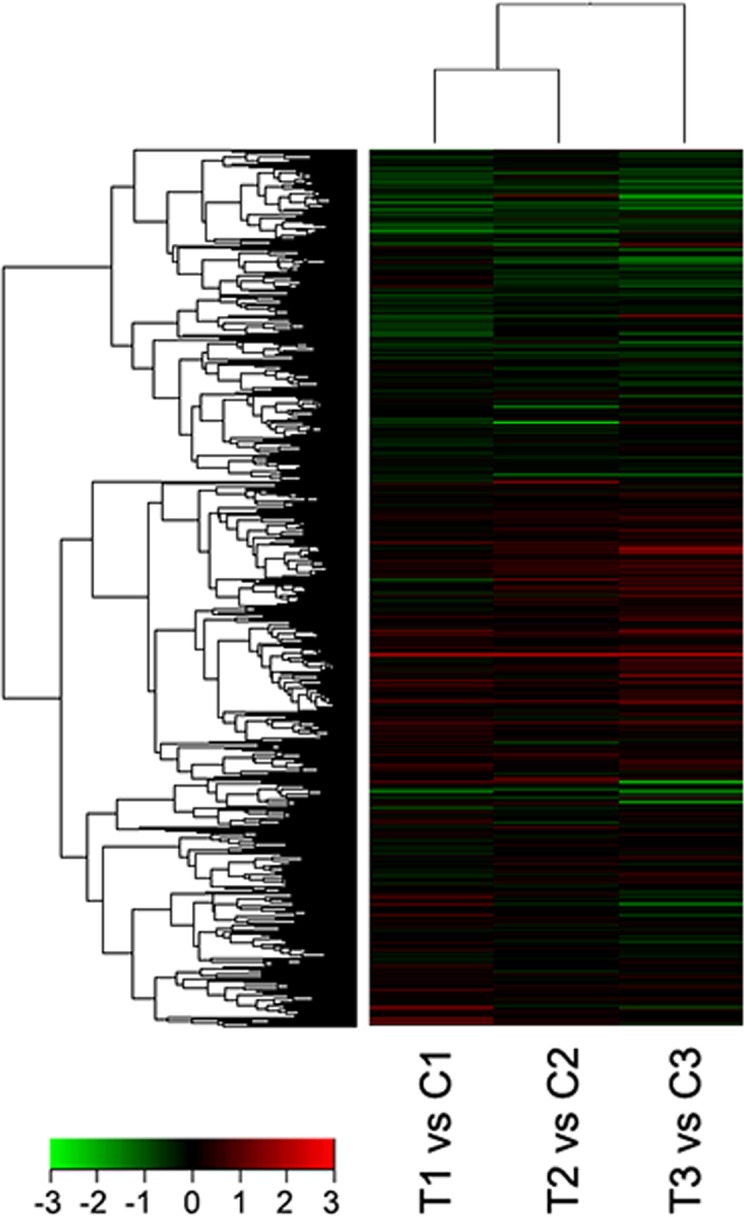
Hierarchical cluster analysis of the data from the different skin samples of goats raised under short and natural photoperiods. The color legend is at the bottom of the Fig Red indicates the genes with greater expression relative to the geometric means, and green indicates the genes with lower expression relative to the geometric means. T1, T2, and T3 represent the 3 samples of side skin from goats raised under the short photoperiod, and C1, C2, and C3 represent the 3 samples of skin from goats raised under the natural photoperiod.

We used the G2519F (Agilent) sheep gene expression profile chip (8 ×15k), which contains 15,208 sheep genes, to analyze differences in the expression patterns of the protein-coding genes in the Cashmere goat hair follicles. We compared the results of the skin samples from the short photoperiod and natural conditions using the hierarchical clustering method in Cluster 3.0 software (Fig 2). The up-regulated genes were highly expressed in the experimental group (red), and the expression levels of the down-regulated genes were significantly decreased (green). The cluster analysis showed that the short photoperiod induces differences in gene expression in Cashmere goat skin.

### Biological process gene ontology (GO) analyses

**Fig 3:**
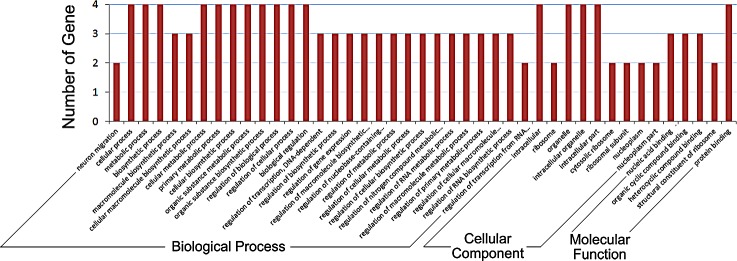
GO classifications for the differentially expressed genes. Differentially expressed genes enriched in the following GO terms are indicated: biological processes, cellular components, and molecular function.

We screened out 112 genes that were significantly differentially expressed (fold change ≥ 2 or ≤ 0.5), of which 63 genes were up-regulated (≥2) and 49 genes were down-regulated (≤0.5). Based on the gene annotations in the database, we attempted to correlate the differentially expressed genes with the regulation of the goat hair follicle. The GO results showed that the genes expressed in the Cashmere goats from the short photoperiod were functionally classified into three main ontologies: biological process, cellular component and molecular function (Fig 3). In the biological process category, the largest relative number of terms annotated to the contigs included the cellular process, metabolic process, biosynthetic process, macromolecule biosynthetic process and related regulation of the macromolecule biosynthetic process.

### Results of the differentially expressed gene analysis

Because of the limitations of these results and the existing information, most of the genes have not been annotated. Based on the existing reports, we screened out 24 genes, 14 up-regulated and 10 down-regulated (Tables 3 and 4), that may be involved in the different stages of hair follicle growth and hair shaft fiber synthesis. Among these, some important genes are associated with hair follicle structure formation, such as keratin and the keratin-associated proteins keratin 27, keratin 34, and keratin 85. In addition to keratin, the cytoskeleton significantly contributes to the formation of the hair follicle, including the type I wool keratin intermediate filament and the type I keratin intermediate filament (Table 3). In addition, many genes, such as cytochrome P450, major histocompatibility complex (MHC) class I antigen, somatostatin receptor, and vascular endothelial growth factor receptor, are not directly involved in the formation of the hair follicle but may be associated with the formation of the accessory structures of the hair follicle, including glands, blood vessels, and other structures, which are vital for the growth of the hair follicle. Taken together, these results show that the short photoperiod induces the differential expression of Cashmere goat hair follicle genes, which affects the natural cyclic activity of the hair follicle, makes the hair follicle enter the anagen phase ahead of schedule, extends the growth cycle of the hair follicle, and ultimately achieves the goal of increased cashmere production.

**Table 3:** Genes associated with the growth cycle of the hair follicle that are up-regulated in the Cashmere goat under the short photoperiod compared with natural conditions.

Gene Symbol	Gene Name	GenBank Accession Number	Fold Change
**MLN**	motilin	NM_001009439	3.5967
**CYP17A1**	"cytochrome P450, family 17, subfamily A, polypeptide 1"	NM_001009483	2.4345
**LOC100125618**	metallothionein-4	NM_001105260	2.406
**V15**	type I wool keratin intermediate filament 8c1	NM_001009445	2.3194
**K27**	keratin 27	NM_001114763	2.3069
**LOC100526781**	keratin 34	HQ283081	2.3041
**SSTR2**	somatostatin receptor 2	AF335550	2.2758
**FLT-1**	vascular endothelial growth factor receptor-1	AF534635	2.2321
**K2.12**	keratin 85	EU216428	2.2016
**LOC443079**	type I keratin intermediate filament IRSa1	NM_001009739	2.0122
**SP-D**	surfactant protein D	AJ133002	2.7986
**TP63**	tumor protein p63	EF491630	2.276
**LAT2**	"linker for activation of T cells family, member 2"	AY162433	2.2725
**JAG1**	jagged 1	DQ152971	2.2057

**Table 4:** Genes associated with the growth cycle of the hair follicle that are down-regulated in the Cashmere goat under the short photoperiod compared with natural conditions.

Gene Symbol	Gene Name	GenBank Accession Number	Fold Change
**FCGR1A**	"Fc fragment of IgG, high affinity Ia, receptor (CD64)"	NM_001139452	0.4764
**OVAR**	MHC class I antigen	NM_001130934	0.4645
**LOC100101238**	regakine 1-like protein	NM_001099629	0.453
**BST-2B**	bone marrow stromal cell antigen 2B	NM_001178051	0.3148
**ISG17**	interferon-stimulated gene 17	NM_001009735	0.1902
**CYP1A1**	cytochrome P4501A1	NM_001129905	0.1034
**SELE**	selectin E	NM_001009749	0.4907
**C5aR**	C5a anaphylatoxin receptor	EE828358	0.487
**ddit3**	DNA damage-inducible transcript 3	FE020981	0.4844
**TLR7**	toll-like receptor 7	NM_001135059	0.4692

### qPCR validation

**Fig 4:**
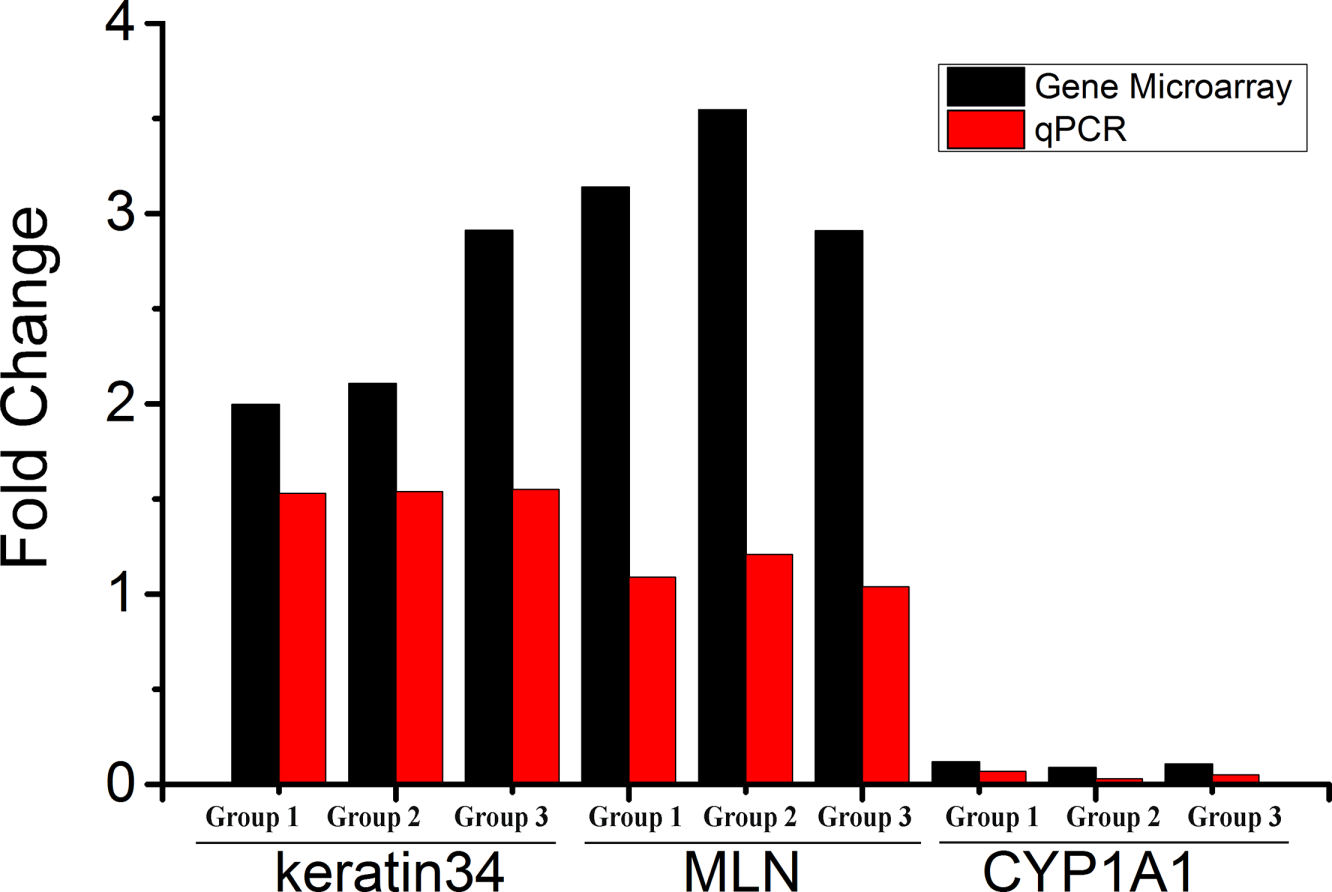
qPCR validation of the expressed genes. For the 3 randomly selected differentially expressed genes, the fold changes of the genes in the short and natural photoperiods were assessed by qPCR (red bar) and compared to those detected in the gene chip microarray (black bar). All Ct values were normalized to GAPDH, and replicates (n = 3) of each sample were run. The P values (T-test) of the Q-PCR data are 0.1096 (keratin 34), 0.0003 (MLN), and 0.0201 (CYP1A1).

To verify the gene chip results, we selected three genes, CYP1A1, keratin 34 and MLN, for qPCR and compared their expression patterns (Fig 4). Although the mRNA expression levels were not significantly different from the gene chip microarray, their trends were highly similar and reflected the reliability of the gene chip analysis results.

## Discussion

### Experimental method and analysis of the results

This study uses a gene chip microarray to study cyclic changes in cashmere growth under manual short photoperiod intervention and compares them to the related gene expression profiles in the hair follicles of Cashmere goats in artificial and natural conditions. All of the samples were obtained in July. Marked differences in gene expression can be observed between the two conditions at this time point, when the secondary follicles of the Cashmere goat have not entered the anagen phase. Therefore, the impact of the original cyclic growth of the hair follicle can be ruled out, and we can ensure that these differences in gene expression are highly reliable. In addition, it is common to study hair follicle-associated gene expression by analyzing skin samples. However, other parts of the skin, in addition to the hair follicles, might influence the gene expression specificity; therefore, in future studies, our analysis of the gene expression results must be more precise to eliminate as much interference as possible.

Due to the limitations of the reproductive characteristics among Cashmere goats, we cannot obtain multiple individuals who have precisely the same genetic background. To ensure the accuracy of our tests, we chose Cashmere goats of the same gender and of similar ages and nutritional conditions, and each Cashmere goat bred in the short photoperiod was compared to a fellow individual (of the same parent) under natural conditions. Thus, we ensured the accuracy and reliability of the differences in gene expression to the extent feasible. In addition, we chose three representative genes for qPCR, and the results were consistent with the microarray data, further demonstrating the reliability of our results.

In this study, we analyzed the molecular activity related to hair follicle growth. Microarray technology helped us to identify genes with greater than 2-fold differences in expression between the two groups, and compared to the complete microarray data, the number of these genes was greatly diminished (112/15,208), which narrows the scope of the relevant genes for hair follicle activity. We also verified the results though qPCR.

### Differences in the expression of genes related to hair structure formation

The most obvious among these differentially expressed genes were those related to keratin protein and keratin-associated protein expression. The hair of the Cashmere goat is diversified into two main types, wool and cashmere, which exists together in the hair follicle group and are produced by the primary and secondary follicles, respectively. The two types are highly similar in terms of structure and composition; in general, wool has a medulla, whereas cashmere does not. Therefore, notable differences in fineness are present, which are correspondingly determined by the shape and size of the hair follicle. Hair growth regulation depends on the cyclic activities of the hair follicle and the successive catagen and telogen phases in the growth cycles [28]. In the anagen phase, cashmere rapidly grows under the control of related genes; keratin protein gene expression is the strongest at this time.

A previous study comparing human hair and different cashmere varieties identified significant differences in the high glycine tyrosine (HGT) keratin-associated protein (KAP) compositions of different animal wools; in particular, the HGT KAP (KAP6, KAP7 and KAP8) contents are less than 3% in human hair, 19% in sheep wool, and up to 30–40% in monotreme mammals [29]. The study also showed that in different species, the changes in the HGT KAP contents were closely related to hair quality. In addition, the studies of Jin et al. [30] have shown that KAP7.1 is a part of the cortex and inner root sheath structures, is involved in the formation of the inner root sheath of cashmere and keratinization of the hair shaft, and plays an important role in the growth of cashmere hair. Meanwhile, KAP7.1 is also expressed in the matrix of the secondary hair follicle. In humans, sheep and other mammals, KAP7.1 is not expressed in the outer root sheath (ORS), sebaceous glands or hair medulla but is expressed in the inner root sheath in both the primary and secondary hair follicles, which may be the reason for the differences in cashmere characteristics among different species [30]. Consistent with this hypothesis, research in Australian Merino sheep also concluded that HGT KAP expression is significantly reduced in mutants, which further indicates that the HGT KAPs can control the quality of the wool fiber [31]. In this experiment, the analysis of the Arbas Cashmere goat microarray revealed the up-regulated expression of keratin 27, keratin 34, keratin 85, KAP6 (EE752086) and KAP7 (EE757001), suggesting that under these experimental conditions, the Cashmere goat hair follicles enter the anagen phase early, inducing increased cashmere production. In addition, we also found significant expression of keratin 85. Although its role in the growth of cashmere is unclear, the hair matrix in the human hair follicle hair shaft is rich in keratin 85 [32], and mutations in the keratin 85 gene can lead to developmental disorders of the hair follicle and hair follicle appendages [33]. These results suggest that this structure plays an important role in hair structure; thus, keratin 85 expression likely indicates that the hair follicle has entered the anagen phase.

Type I and type II keratin intermediate filaments (KIFs) are coordinately expressed in the different parts of each hair follicle and are expressed with filament-associated proteins to form the structures that promote the growth of the hair follicle and fiber synthesis. Research has demonstrated that many epidermal cytokeratin pairs are expressed in a specific hair follicle cell layer [34].

Keratin is present throughout the epithelial tissue, and intermediate filaments are primarily composed of this protein and constitute the cytoskeletons of mammalian cells [35]. Tzu-Hao Chang et al. [36] found that KRT14, KRT19, KRT34, KRT-associated proteins (KRTAP) 1 to 5, and KRTAP2-3 were involved in the clustering of keratin (KRT) intermediate filaments. These genes are located on chromosome 17q21.2 and cause the formation of the actin cytoskeleton [36]. Therefore, the keratin intermediate filaments and the expression of the relevant downstream keratin genes are vital for the formation of the hair shaft. Thus, we can presume that these genes will be extremely active when the hair follicles enter the anagen phase, as shown in our study. Upon analysis of the microarray data, V15 (type I wool keratin intermediate filament eight c1), LOC443079 (type I keratin intermediate filament IRSa1) and the downstream genes LOC100526781 and keratin (34) were significantly up-regulated in the skin samples of the Cashmere goats in the experimental group, indicating that the genes that construct the hair shaft are highly active. These results provide important evidence that the shorter photoperiod can induce hair follicle growth.

### Differences in the expression of genes related to the hair follicle peripheral skin appendages

Immune privilege (IP) occurs in the anagen phase [22]. In the hair bulb and hair follicle bulge, the down-regulation of MHC class I is always accompanied by the expression of immunosuppressive factors. Previous studies have found that class I and class II human leukocyte antigen (HLA) alleles are significantly down-regulated in the hair bulb and root sheath, and this down-regulation is accompanied by the up-regulation of a variety of immunoregulatory genes. Compared to the epidermis, SST (somatostatin) is significantly up-regulated in these tissues. SST expression is strongest in the outer root sheath of the hair follicle, and cultures of sheath cells in vitro also secrete SST [37]. Our results showed that SSTR2 (somatostatin receptor 2) was significantly up-regulated in the skin samples of the experimental group, whereas OVAR (an MHC class I antigen) expression was significantly down-regulated. These findings are in accordance with previous reports and demonstrate that the hair follicles have entered the anagen phase; accordingly, cashmere starts to grow.

The relationship between the CYP (cytochrome P450) gene families and the control of hair follicle growth has been reported in numerous studies [38, 39]. CYP1A1 (cytochrome P450 1A1) is expressed at a low level in mouse skin; in contrast, it is expressed at a higher level in the hair follicle sebaceous glands [40]. Studies in goats have indicated that the expression of CYP17A1 (cytochrome P450 17A1) causes hypocortisolism in Angora goats in South Africa [41]. Our experimental results indicate that CYP17A1 is significantly up-regulated and that CYP1A1 is significantly down-regulated in the experimental group, which may be related to hair follicle growth and the construction of the hair follicle appendages during the anagen phase. This interesting phenomenon still requires further in-depth research.

Tissue regeneration depends on an adequate blood supply, an important prerequisite of which is the formation of blood vessels, particularly in the early development and formation of the hair follicles [42]. As dermal papillae increase in number and size, more blood is required to transport nutrients and send signals; thus, a rich network of blood vessels is formed around the dermal papillae. During development, vascular endothelial growth factor (VEGF) is involved in regulating blood vessel formation. Moreover, as an important factor in blood vessel formation and regeneration, VEGF is expressed in skin during wound healing and the periodic vascular remodeling of the hair follicle. VEGF expression is particularly high when the dermal papillae are active to prompt blood vessel growth to meet the massive nutritional requirements of the hair follicles during anagen. Therefore, to some extent, VEGF expression indirectly reflects the degree of skin hair follicle growth [43]. It has been confirmed that the controlled release of VEGF in mice can induce cyclic hair follicle growth [44], and up-regulated VEGF expression can also lead to the regional growth of human hair follicles [45]. These studies have demonstrated the importance of VEGF for hair follicle growth and cyclic activities. To date, many studies have reported that VEGF receptor 2 (VEGFR-2) is expressed in the cells of human hair follicles, sebaceous glands, sudoriparous glands and other locations. VEGFR-2 plays an important role in the growth of the hair follicles [46] and is associated with bulge, K15 and K19 expression [47]. However, the role of VEGF in wool growth has been unclear. Our results reveal that the short photoperiod induced differential VEGFR-1 expression in the hair follicles in skin during the anagen phase, which provides important insight that VEGFR-1 may participate in the reconstruction of periodic Cashmere goat hair follicles, a possibility that should be investigated further.

### Differences in the expression of genes related to photoperiod

In Human hair growth model, the seasonal variation is not obvious, hair cycle is usually unsynchronized after age one. Unlike human, Cashmere goat is one of the typical species whose hair cycle is associated with hormone secretion and affected by the environmental change especially light cycle. In one year, the changeable length of sunlight can cause differences in hormone secretion, such as the pineal melatonin, forming a nerve-hormone regulation system to control hair follicle cyclic growth[48].It is surprising that the prolactin receptor (PRLR), responding to the photoperiod signal PRL in mammals, is expressed similarly in the two groups. In the Australian Cashmere goat, which exhibits seasonally dependent cycles of pelage, the increasing PRL during spring was shown to reactivate telogen follicles and induce them into the anagen phase [49]. Although PRL and melatonin have been shown to stimulate hair shaft elongation in vitro in Cashmere goats [50, 51], Wiltshire sheep show increasing PRL levels after experimentally extended photoperiods associated with a short-term inhibitory effect on growing anagen follicles [52, 53]. This phenomenon is consistent with the Arbas Cashmere goat, in which the reactivity and subsequent inhibitory effect of secondary hair follicles are observed in growing proanagen follicles. Although PRL was not detected in this study, PRLR mRNA has been reported in the dermal papilla and outer root sheath of ovine wool follicles and showed cycle-related patterns, indicating the dynamic regulation of PRLR by prolactin, which modulates the hormonal responsiveness of seasonally growing hair follicles [53]. This microarray chip contains the PRLR mRNA probe, but no differences were noted between the experimental and control groups. This lack of difference may have occurred because this method is not sufficiently sensitive or because PRLR is actually identical in the two groups. Indeed, at this stage, secondary hair follicles have been in the proanagen phase for up to three months. The short photoperiod can shorten the proanagen phase and promote the entrance of secondary hair follicles into anagen immediately, resulting in fast-growing cashmere.

## Conclusions

A comparative study of histology and gene expression in Cashmere goat skin samples exposed to an artificial short photoperiod and natural conditions demonstrated that a short photoperiod causes the Cashmere goat hair follicles to enter the anagen phase early. The gene chip data demonstrate that differential gene expression is involved in hair follicle growth, hair shaft structure formation, and biological processes of the skin appendages; in particular, the active expression of keratin and cytoskeleton-associated genes during this period provides important evidence of hair shaft formation. The results reveal that photoperiod is the main factor affecting the growth of cashmere; under the short photoperiod conditions, genes involved in the hair follicle growth process will be mobilized to participate in cyclic hair follicle growth. Controlling the light cycle is an effective method for increasing the production of cashmere and can be widely used in actual livestock production.
